# Gualou Guizhi Granule Suppresses LPS-Induced Inflammatory Response of Microglia and Protects against Microglia-Mediated Neurotoxicity in HT-22 via Akt/NF-*κ*B Signaling Pathways

**DOI:** 10.1155/2021/9957459

**Published:** 2021-07-22

**Authors:** Xiaona Chang, Yaling Fang, Yuqin Zhang, Yaojun Liu, Liming Fan, Lihong Nan, Wei Xu, Yu Lin, Kedan Chu, Guohong Yan

**Affiliations:** ^1^Pharmacy College of Fujian University of Traditional Chinese Medicine, Shangjie Minhou, Fuzhou, Fujian, China; ^2^State Key Laboratory of Chinese Pharmacies of Fujian Provincial Department of Science and Technology of Fujian University of Traditional Chinese Medicine, Shangjie Minhou, Fuzhou, Fujian, China; ^3^People's Hospital Affiliated of Fujian University of Traditional Chinese Medicine, Fuzhou, Fujian, China

## Abstract

Neuroinflammation plays a crucial part in the commencement and advancement of ischemic stroke. Gualou Guizhi granule (GLGZG) is known to well exhibit neuroprotective effect, but it is not known whether GLGZG can regulate the inflammatory process at the cellular level in BV2 microglia cells and protect against microglia-mediated neurotoxicity in neurons. Herein, we aimed to investigate the anti-inflammatory effects of GLGZG on BV2 microglia cells and protection against microglia-mediated neurotoxicity in neurons. *Methods*. The cell model of neuroinflammation was constructed by lipopolysaccharide (LPS) to observe the effect of GLGZG in the presence or absence of GLGZG. The production of nitric oxide (NO), inflammatory mediators, was detected. Moreover, potential mechanisms associated with the anti-inflammatory effect, such as inhibition of microglial activation and nuclear factor kappa B (NF-*κ*B), were also investigated. In addition, to prove whether GLGZG protects against microglia-mediated neurotoxicity, neuronal HT-22 cells were cultured in the conditioned medium. And cell survivability and neuronal apoptosis of HT-22 were evaluated. *Results*. It was found that a main regulator of inflammation, NO, is suppressed by GLGZG in BV2 microglial cells. Moreover, GLGZG dose dependently decreased the mRNA and protein levels of inducible NO synthase (iNOS) in LPS-stimulated BV2 cells. Additionally, GLGZG inhibited the expression and secretion of proinflammatory cytokines in BV2 microglial cells. Also, GLGZG inhibited LPS-activated nuclear factor kappa-light-chain-enhancer of activated B cells (NF-*κ*B) in BV2 microglial cells at the intracellular level. GLGZG significantly affected Akt phosphorylation: phosphorylated forms of Akt increased. To check whether GLGZG protects against microglia-mediated neurotoxicity, neuronal HT-22 cells were incubated in the conditioned medium. GLGZG showed a neuroprotective effect by promoting cell survivability and suppressing neuronal apoptosis. *Conclusions*. GLGZG exerted its potential effects on suppressing inflammatory responses in LPS-induced BV2 cells by regulating NF-*κ*B and Akt pathways. In addition, GLGZG could protect against microglia-mediated neurotoxicity in HT-22.

## 1. Introduction

Stroke, especially ischemic stroke, is a life-threatening disease which is considered the most common cause of disability and mortality around the world [1, 2]. Although ischemic stroke involves the interaction of numerous pathophysiological processes, accumulating evidence proves that neuroinflammation plays an important role in the pathological process involved in ischemic stroke [3–6]. Recent studies have proved that microglia are critically involved in regulating neuroinflammation [7].

Microglia, as the main immune cells in the brain, act as the first major safeguard of the central nervous system (CNS). In the physiological state, microglia are at the resting state; they survey the CNS microenvironment via continuously extending and retracting ramified processes, while they were activated in a number of neurodegenerative diseases and different types of brain injury [8–10]. Ischemic stroke is a powerful stimulus that triggers microglial activation. Once activated, microglia develop macrophage-like capabilities and polarize into M1 or M2 phenotype. They are considered as a double-edged sword [11, 12]. M2 microglia are regarded as “healing cells,” and they clear debris, release anti-inflammatory mediators, and function to help repair injury. On the contrary, M1 microglia are considered as proinflammatory, releasing proinflammatory mediators, and function to exacerbate ischemic injury; at the same time, peripheral leukocytes infiltrate into the brain, and the normally immune-privileged brain environment is exposed to systemic responses that further exacerbate inflammation and brain damage, resulting in a vicious cycle. Thus, regulating microglial phenotype might be a potential treatment for neuroinflammation of ischemic stroke.

It is proved that the activated microglia-mediated neuroinflammation is mainly regulated by transcription factor nuclear factor kappa B (NF-*κ*B), a crucial modulator of various kinds of inflammation [13]. As we know, NF-*κ*B is bound to the inhibitor of kappa B (I*κ*B) proteins in the cytosol which is inactive. And when I*κ*B kinase (IKK) first phosphorylates and degrades I*κ*B, NF-*κ*B begins to be activated; NF-*κ*B is dissociated from the complex and transferred to the nucleus. Then, it activates the transcription of downstream genes and regulates the role of inflammatory mediators synthesis and release. Furthermore, the phosphatidylinositol 3-kinase/Akt (PI3K/Akt) signaling pathway is also involved in NF-*κ*B activation and proinflammatory responses [14].

Chinese classical prescriptions are important resources to develop safe and effective candidates for neuroinflammation of ischemic stroke. Gualou Guizhi granule (GLGZG, Min drug system approval no. S20130001) is a standard hospital prescription at the Fujian University of TCM Affiliated Second People's Hospital (Fuzhou, China), which was first reported by Zhang et al.[15]. It consists of 6 kinds of Chinese herbs: *Trichosanthes kirilowii* Maxim., *Cinnamomum cassia* Presl., *Paeonia lactiflora* Pall., *Zingiber officinale* Rosc., *Ziziphus jujuba* Mill., and *Glycyrrhiza uralensis* Fisch. It has long been used in clinics to treat muscular spasticity and dyskinesia following stroke, epilepsy, or spinal cord injury in China with a weight ratio of 10 : 3 : 3 : 3 : 2 : 3 [16–21].

Moreover, in recent years, studies have also documented that GLGZG well exerts effects of anti-inflammation, anticerebral ischemia-reperfusion injury, and neuroprotection [22–27]. Although preliminary research studies suggested a potential relationship between GLGZG and neuroinflammation, whether it can regulate the inflammatory process at the cellular level in BV2 microglia cells and protect against microglia-mediated neurotoxicity in neurons and the underlying mechanisms have not been well investigated. In the current study, we aimed to investigate the anti-neuroinflammatory effects of GLGZG in LPS-stimulated BV2 microglial cells. Furthermore, the roles of GLGZG in the Akt/NF-*κ*B-related signaling pathway were investigated.

## 2. Materials

GLGZG was presented by the Pharmaceutical Department of Fujian University of Traditional Chinese Medicine Affiliated Second People's Hospital (Fuzhou, China). It was certified and standardized on the basis of labeled compounds (the Food and Drug Administration in Fujian Province, 2013). Our phytochemical studies also illustrated that 104 compounds in GLGZG and several bioactive components, such as citrulline, luteolin, puerarin, liquiritin, taxifolin, naringin, formononetin, isoliquiritigenin, 6-gingerol, curcumin, caffeic acid, ferulic acid, jujuboside A, protocatechuic acid, cinnamic acid, catechin, and paeoniflorin, were identified or tentatively characterized [28–30]. Lipopolysaccharide (LPS, *Escherichia coli* serotype 055:B5) was purchased from Sigma (St. Louis, MO). RPMI Medium 1640 basic and fetal bovine serum (FBS) were purchased from Thermo Fisher Scientific (Carlsbad, CA, USA). CellTiter 96^®^ AQueous One Solution Cell Proliferation Assay was bought from Promega (Madison, USA). IL-1*β*, IL-6, IL-10, and TNF-*α* mouse ELISA kits were obtained from ABclonal (Wuhan) Biotechnology Co. Ltd., Wuhan, China. Rabbit or mouse antibodies against Akt, p-Akt (Ser473), PI3K (p85), PI3K (p110*α*), IKK*β*, p-I*κ*B*α* (Ser32/36), I*κ*B*α*, NF-*κ*Bp65, and *β*-actin were purchased from Cell Signaling Technology (Boston, MA, USA). The secondary antibodies conjugated with horseradish peroxidase (HRP) were all bought from Xiamen Lulong Biotech Co., Ltd. (Xiamen, China).

## 3. Methods

### 3.1. Cell Culture and Treatment

BV2 microglial cells and neuronal HT-22 were obtained from Beijing Beina Chuanglian Biotechnology Research Institute (Beijing, China). In brief, cells were cultured in the RPMI 1640 medium with 10% FBS and 1% penicillin-streptomycin mixed solution (Gibco Invitrogen Corporation, Carlsbad, CA, USA) at 37°C in a 5% CO_2_ incubator.

In these studies, the cells were seeded on 96-well plates or 6-well plates and were divided into three groups randomly: (1) pure BV2 cell group as the control group (BV2 group); (2) LPS group, where BV2 cells were incubated with LPS (1 *μ*g/mL); and (3) LPS plus GLGZG (50, 100, and 200 *µ*g/mL) group, where cells were coincubated with LPS (1 *μ*g/mL) and GLGZG (50, 100, and 200 *µ*g/mL) for 24 h. Culture supernatants were harvested for ELISA experiments and nitric oxide assay. Additionally, the cells were then harvested for RNA or protein isolation.

To determine the protective effect of GLGZG against microglia-mediated neurotoxicity, neuronal HT-22 cells were incubated in the conditioned medium from the control, LPS, and LPS plus GLGZG groups of BV2 cells. Conditioned media were then tested for cell viability using CellTiter 96^®^ AQueous One Solution Cell Proliferation and LDH assays, and cell lysates were used to test for apoptosis from conditioned media-treated HT-22 cells.

### 3.2. Cell Viability Assay

The cultured cells were treated with LPS with or without GLGZG for 24 h. After treatment, the cell viability was assessed by CellTiter 96^®^ AQueous One Solution Cell Proliferation Assay. CellTiter 96^®^ AQueous one solution was added and incubated for another 4 h at 37°C. Then, the absorbance at 570 nm was taken by using a microplate reader (Infinite M200 Pro, TECAN). The cell viability was calculated according to the OD value. Survival rate (%) = ODexperimental group/ODcontrol group × 100%.

### 3.3. ELISA Experiments

The cultured cells were treated with LPS with or without GLGZG for 24 h for ELISA. After treatment, culture supernatants were collected and then centrifuged prior to the determination of IL-1*β*, IL-6, IL-10, and TNF-*α* production. Detailed manipulation process was performed by manufacturer protocols of mouse ELISA kits.

### 3.4. Nitric Oxide Assay

The cultured cells were treated with LPS with or without GLGZG for 24 h for NO assay. After treatment, the culture supernatants were collected, and NO production was measured by assessing the nitrite level in the culture media. It was executed by mixing the medium with Griess reagent. Optical concentration was analyzed at 540 nm after 10 minutes of incubation.

### 3.5. Quantitative Real-Time PCR Analysis

The cultured cells were treated with LPS with or without GLGZG for 6 h for quantitative real-time PCR. After treatment, cells were washed with PBS, total RNA was extracted by RNeasy^®^ Mini Kit, and the RNA concentration was determined. Then, RevertAid First Strand cDNA Synthesis Kit was used for reverse transcription to cDNA. The cDNA product was used as a template for quantitative PCR amplification, and it was then carried out on ABI 7900HT Real-Time PCR System (Applied Biosystems Inc., Foster City, CA, USA). The data were analyzed by the 2^−△△CT^ relative quantification method. The relative transcriptional level of target genes normalized to GAPDH was calculated. The primer sequences for the amplification of the target genes are shown in Table 1.

### 3.6. Western Blot Analysis

The cultured cells were treated with LPS with or without GLGZG for 6 h for western blotting for relative protein expression. The cells were inoculated into a 6-well plate with a density of 3 × 10^5^/well and cultured for 24 h and then treated with indicated concentrations of GLGZG and LPS (1 *μ*g/mL) for 24 h. Cells were collected and lysed by RIPA lysis buffer, and then they were centrifuged at 12,000*g* for 15 min. The supernatant was collected, and the protein concentration was determined by the BCA method. Then, protein was mixed with loading buffer and incubated at 100°C for 6 min. Ultimately, samples were analyzed for western blot analysis with primary antibodies to iNOS (1 : 1000), CD206 (1 : 1000), p-Akt (Ser473) (1 : 2000), Akt (1 : 1000), PI3K (p85) (1 : 1000), PI3K (p110*α*) (1 : 1000), IKK*β* (1 : 1000), p-I*κ*B*α* (1 : 1000), I*κ*B*α* (1 : 1000), NF-*κ*Bp65 (1 : 1000), Bax (1 : 1000), Bcl-2 (1 : 1000), NeuN (1 : 1000), and *β*-actin (1 : 1000) overnight at 4°C followed by incubating with the horseradish peroxidase-conjugated secondary antibody IgG (HRP Goat Anti-Rabbit IgG secondary antibody (1 : 5000) and Goat Anti-mouse IgG secondary antibody (1 : 5000), Lulong Biotech Co., Xiamen, China) at room temperature. Finally, they were evaluated using the ECL western blotting detection reagents, and the relative expression level of target genes normalized to *β*-actin was analyzed.

### 3.7. Immunofluorescence Assay

Immunofluorescence assay was performed to detect the nuclear translocation of the NF-kBp65 subunit. In brief, cells were fixed using 4% paraformaldehyde for 15 min at room temperature, followed by washing with PBS for 5 min 3 times. The cells were blocked with 5% BSA prepared in 0.1% Tween 20 (PBST) and 0.3% Triton X-100 for 1 h at room temperature. Afterward, cells were incubated with NF-*κ*Bp65-specific primary antibody (1 : 100, Cell Signaling Technology, MA, USA) overnight at 4°C. After washing, cells were incubated with the FITC-labeled IgG secondary antibody (Beijing Zhongshan Jinqiao Biotechnology Co. Ltd., Beijing, China, 1 : 200 dilution in 5% BSA solution) for 1 h in dark. After that, cells were stained with 25 *μ*g/mL of 4′-6-diamidino-2-phenylindole (DAPI) in PBST. Finally, samples were documented using the Olympus IX73 fluorescence microscope (Tokyo, Japan).

### 3.8. Statistical Analysis

All the experiments were repeated three times. The data were statistically analyzed by SPSS 19.0 software and expressed as mean ± standard deviation ($\bar{x}$ ± *s*). The difference between groups was analyzed by one-way ANOVA followed by the Student–Newman–Keuls *q*-test with post hoc correction which was employed for multiple comparisons, and *P* < 0.05 was considered to indicate statistical significance.

## 4. Results

### 4.1. GLGZG Modulated Expressions of LPS-Induced Microglia Activation

**Figure 1:**
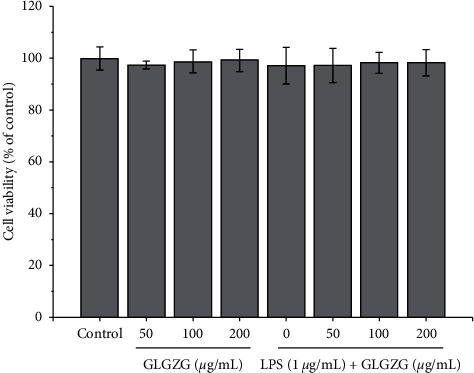
GLGZG in the presence and absence of LPS did not have a significant effect on microglial viability.

According to the past research result, 50, 100, and 200 *μ*g/mL of GLGZG were employed in these experiments [26]. As illustrated in Figure 1, GLGZG in the presence and absence of LPS did not have a significant effect on microglial viability.

**Figure 2:**
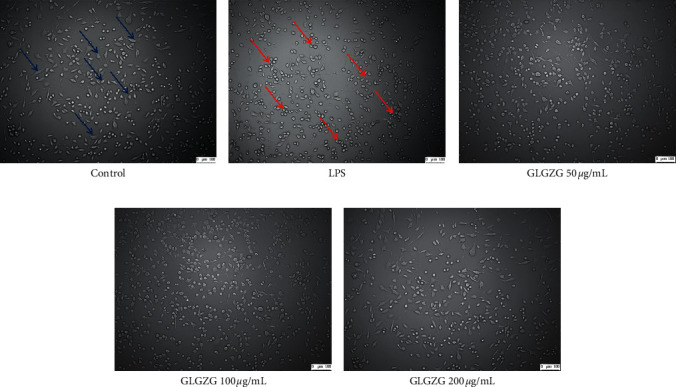
The LPS-activated cells were characterized by round and an absence of thin processes, which was considered as an “active stage” phenotype, while as compared to LPS-activated cells, the control and GLGZG-treated LPS-activated BV2 cells showed less rounded and more elongated cells with thinner processes, which was considered as “resting stage” phenotype.

Morphological examination result is presented in representative photomicrographs in Figure 2. As shown in Figure 2, the LPS-activated cells were characterized by round and an absence of thin processes, which was considered as an “active stage” phenotype, while as compared to LPS-activated cells, the control and GLGZG-treated LPS-activated BV2 cells showed less rounded and more elongated cells with thinner processes, which is considered as “resting stage” phenotype.

**Figure 3:**
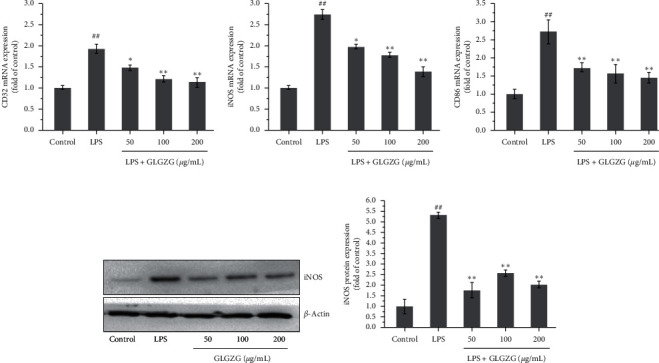
Expression of markers of LPS-activated cells in the culture was determined by real-time PCR and western blot analysis of the marker iNOS. (a–c) LPS treatment caused a significant increase in mRNA levels of three markers: iNOS (*P* < 0.01), CD32 (*P* < 0.01), and CD86 (*P* < 0.05), and GLGZG treatment obviously attenuated the effect for iNOS (*P* < 0.05 and *P* < 0.01), CD32 (*P* < 0.05 and *P* < 0.01), and CD86 (*P* < 0.05 and *P* < 0.01). (d, e) The protein expression of iNOS is significantly upregulated after the LPS activation of BV2 microglia cells (*P* < 0.01), while this effect is obviously attenuated by GLGZG treatment (*P* < 0.05).

**Figure 4:**
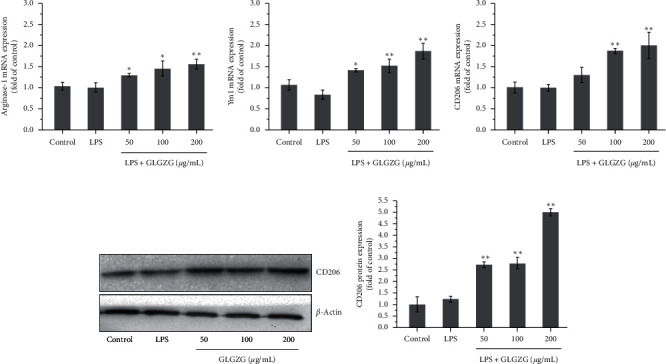
mRNA expression of three M2 markers and the protein expression of the M2 marker CD206 were examined. (a–c) LPS treatment had no significant effect on CD206, arginase-1, and Ym1, three M2 markers; however, GLGZG treatment significantly elevated the expression for CD206, arginase-1, and Ym1 (*P* < 0.05 and *P* < 0.01). (d, e) The protein result of CD206 revealed that LPS had no significant effect on CD206 protein, while GLGZG treatment significantly upregulated CD206 protein.

To further confirm these findings, expression of markers of LPS-activated cells in the culture was determined by real-time PCR. As shown in Figures 3(a)–3(c), LPS treatment caused a significant increase in mRNA levels of three markers: iNOS (*P* < 0.01), CD32 (*P* < 0.01), and CD86 (*P* < 0.05), and GLGZG treatment obviously attenuated the effect for iNOS (*P* < 0.05 and *P* < 0.01), CD32 (*P* < 0.05 and *P* < 0.01), and CD86 (*P* < 0.05 and *P* < 0.01). Western blot analysis of the marker iNOS (Figures 3(d) and 3(e)) indicates that the protein expression of iNOS is significantly upregulated after the LPS activation of BV2 microglia cells (*P* < 0.01, Figures 3(d) and 3(e)), while this effect is obviously attenuated by GLGZG treatment (*P* < 0.05). Likewise, mRNA expression of three M2 markers and the protein expression of the M2 marker CD206 were examined. As shown in Figure 4, LPS treatment has no significant effect on CD206, arginase-1, and Ym1, three M2 markers; however, GLGZG treatment significantly elevated the expression for CD206, arginase-1, and Ym1 (*P* < 0.05 and *P* < 0.01). The protein result of CD206 revealed that LPS had no significant effect on CD206 protein, while GLGZG treatment significantly upregulated CD206 protein.

### 4.2. GLGZG Suppressed LPS-Induced Inflammatory Response in LPS-Activated BV2 Microglia

**Figure 5:**
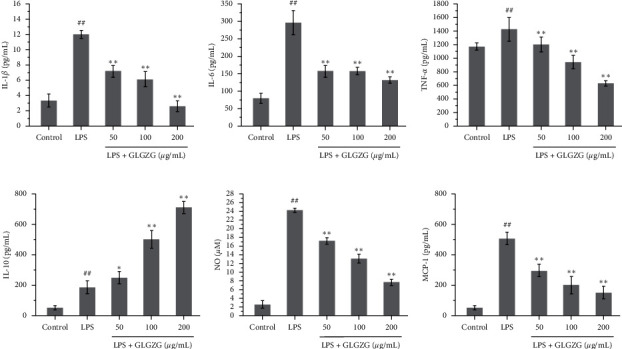
The effect of GLGZG on LPS-induced microglia release, IL-1*β*, IL-6, IL-10, MCP-1, TNF-*α*, and NO. Compared with the control group, the content of IL-1*β*, IL-6, MCP-1, TNF-*α*, and NO in the LPS group increased significantly, while compared with the LPS group, IL-1*β*, IL-6, MCP-1, TNF-*α*, and NO were decreased significantly in the cells of the 50, 100, and 200 *μ*g/mL dose group of GLGZG. In addition, GLGZG also markedly increased the production of IL-10.

To further investigate the effect of GLGZG on neuroinflammation in LPS-activated BV2 microglia, we determined the effect of GLGZG on neuroinflammatory responses, such as cytokines including IL-1*β*, IL-6, IL-10, MCP-1, and TNF-*α* and inflammatory mediators including NO. The contents of IL-1*β*, IL-6, IL-10, MCP-1, TNF-*α* and NO are shown in Figure 5. Compared with the control group, the content of IL-1*β*, IL-6, MCP-1, TNF-*α*, and NO in the LPS group increased significantly, while compared with the LPS group, IL-1*β*, IL-6, MCP-1, TNF-*α*, and NO were decreased significantly in the cells of the 50, 100, and 200 *μ*g/mL dose group of GLGZG. In addition, GLGZG also markedly increased the production of IL-10.

**Figure 6:**
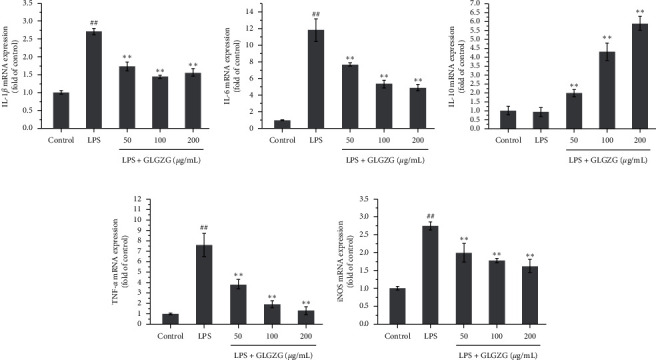
Effects of GLGZG on the mRNA expression of IL-1*β*, IL-6, IL-10, TNF-*α*, and iNOS. GLGZG not only inhibited LPS-induced NO production, iNOS, IL-1*β*, IL-6, IL-10, and TNF-*α* but also significantly reduced the mRNA expression of them.

Moreover, the mRNA expression of IL-1*β*, IL-6, TNF-*α*, and iNOS was measured, all of which were inhibited by treatment with GLGZG (Figure 6). These results suggest that GLGZG not only inhibits LPS-induced NO production, iNOS, IL-1*β*, IL-6, IL-10, and TNF-*α* but also significantly reduces the mRNA expression of them.

### 4.3. GLGZG Inhibited the Activation of the NF-*κ*B Pathway in LPS-Activated BV2 Microglia

**Figure 7:**
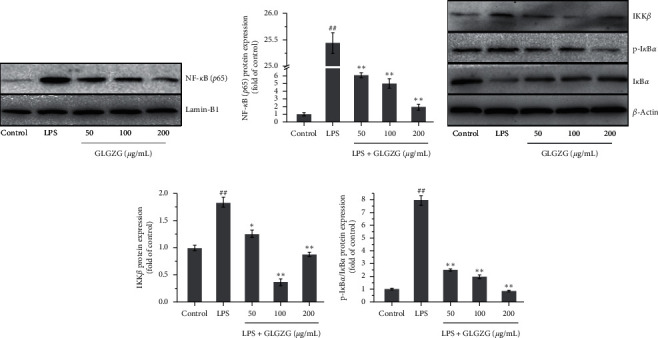
GLGZG inhibited the activation of the NF-*κ*B pathway in LPS-activated BV2 microglia. (a, b) GLGZG dose dependently suppressed NF-*κ*Bp65 expression in the nucleus compared with the average levels in the LPS group (*P* < 0.01). (c, d) Effects of GLGZG on the protein expression of regulators of NF-*κ*B in the cytosol. I*κ*B*α* expression decreased and increased with GLGZG treatment, while p-I*κ*B*α* and IKK*β* expression, respectively, increased and decreased with GLGZG treatment compared to the LPS group (*P* < 0.01).

As shown in Figures 7(a)–7(e), GLGZG dose dependently suppressed NF-*κ*Bp65 expression in the nucleus compared with the average levels in the LPS group (*P* < 0.01). And to confirm the reduced nuclear translocation, regulators of NF-*κ*B in the cytosol, such as I*κ*B, p-I*κ*B, and IKK*β*, were investigated. As expected, I*κ*B*α* expression decreased and increased with GLGZG treatment, while p-I*κ*B*α* and IKK*β* expression, respectively, increased and decreased with GLGZG treatment compared with the LPS group (*P* < 0.01).

**Figure 8:**
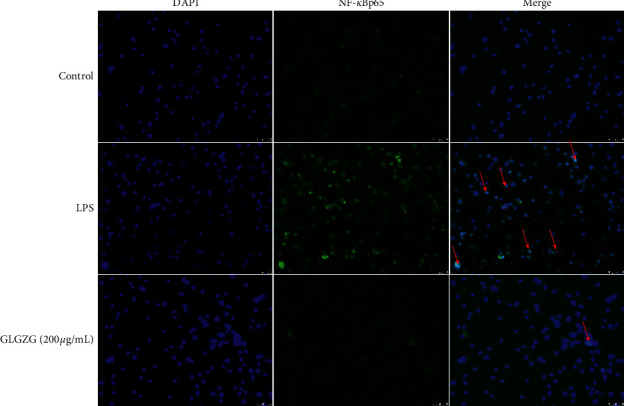
Typical immunohistochemical photographs of NF-*κ*B. NF-*κ*B nuclear translocation in LPS-treated cells reduced with GLGZG treatment (scale bars, 50 *μ*m).

Furthermore, in order to check the decrease in the translocation of the nuclear transcription factor, we directly assayed the intracellular translocation of the factor by immunofluorescence, and NF-*κ*B nuclear translocation in LPS-treated cells reduced with GLGZG treatment, as observed by immunofluorescence microscopic analysis, indicating specific suppression of NF-*κ*B nuclear translocation (Figure 8).

### 4.4. GLGZG Suppressed Akt Phosphorylation in LPS-Activated BV2 Microglia

**Figure 9:**
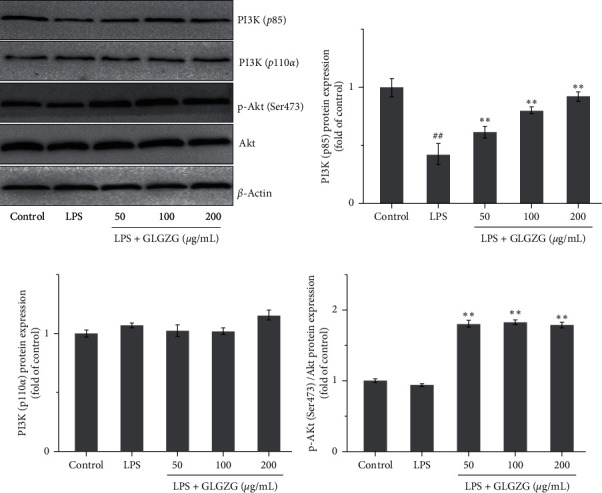
Effects of GLGZG on the protein expression of PI3K (p85), PI3K (p110*α*), Akt, and p-Akt (Ser473). GLGZG could significantly increase the expression of p-Akt (Ser473) and PI3K (p85) compared with the LPS group, while the expression of Akt and PI3K (p110*α*) had no significant change among groups.

As shown in Figure 9, GLGZG could significantly increase the expression of p-Akt (Ser473) and PI3K (p85) compared with the LPS group, while the expression of Akt and PI3K (p110*α*) had no significant change among groups.

### 4.5. GLGZG Protected against Microglia-Mediated Neurotoxicity

**Figure 10:**
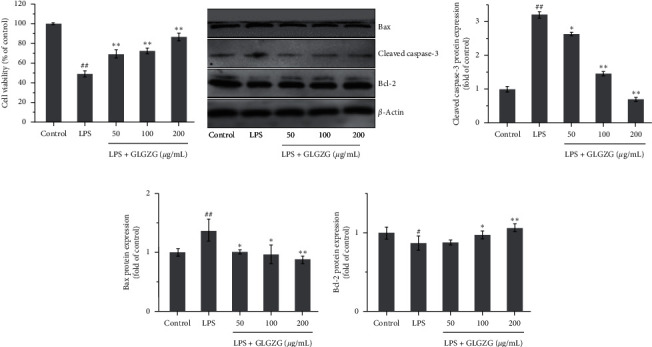
(a) Conditioned medium from LPS plus GLGZG-treated BV2 microglia cells significantly enhanced cell viability (69.56%, 72.13%, and 86.34%) on HT-22 cells, as compared to the LPS-treated BV2 microglia cell conditioned medium (*P* < 0.01). Effects of GLGZG on the protein expression of Bcl-2, cleaved caspase-3, and Bax. (b–e) The levels of Bcl-2 notably declined, while cleaved caspase-3 and Bax increased in the LPS-treated BV2 microglia cell conditioned medium. However, GLGZG exhibited a more pronounced effect on Bcl-2, cleaved caspase-3, and Bax expression.

As shown in Figure 10(a), the results show that the conditioned medium from LPS plus GLGZG-treated BV2 microglia cells significantly enhanced cell viability (69.56%, 72.13%, and 86.34%) on HT-22 cells, as compared to the LPS-treated BV2 microglia cell conditioned medium (*P* < 0.01). Furthermore, as shown in Figures 10(b)–10(e), the levels of Bcl-2 notably declined, while cleaved caspase-3 and Bax increased in the LPS-treated BV2 microglia cell conditioned medium. However, GLGZG exhibited a more pronounced effect on Bcl-2, cleaved caspase-3, and Bax expression.

## 5. Discussion

Neuroinflammatory responses are inevitable and vital pathological processes in ischemic stroke, and microglial activation-mediated inflammatory responses play an important role in brain neuroinflammation and subsequent neuronal injuries. Activated microglia can be characterized by M1 (proinflammatory) and M2 (neuroprotective/anti-inflammatory) phenotypes [31]. BV2 cells stimulated by LPS could provide a very reproducible and robust model for the induction of the M1 phenotype and an inflammatory activation profile of BV2 microglia cells, and it is highly similar to the activation of primary microglia [32, 33]. Since GLGZG was found to be anti-inflammatory and neuroprotective in several reports, in this study, whether GLGZG could act directly upon microglia cells to regulate microglial polarization, proinflammatory and anti-inflammatory cytokine gene expression, and the neurotoxic ability of activated microglia were evaluated in vitro.

Indeed, the results revealed that GLGZG decreased the production of the M1 markers, iNOS, CD32, TNF-*α*, and IL-1*β*. Correspondingly, GLGZG also obviously inhibited the production of the proinflammatory mediators IL-1*β*, IL-4, IL-6, TNF-*α*, and IFN*γ* and increased the level of IL-10 in LPS-activated BV2 microglia cells. As we know, proinflammatory cytokines, e.g., TNF-*α*, IL-6, and IL-1*β*, can promote neuronal damage and also induce more microglia activation as feedback [34]. In addition, some studies indicated that enhanced M2 polarization may be beneficial due, in large part, to a switch of production from M1 “proinflammatory cytokines” to M2 “anti-inflammatory cytokines,” thus decreasing inflammation and facilitating tissue and cellular repair [35, 36]. The findings suggested that GLGZG exerted anti-neuroinflammatory effects by inhibiting BV2 activation.

Moreover, at the intracellular level, GLGZG was also shown to inhibit LPS-activated nuclear translocation of NF-*κ*B in BV2 cells. To our knowledge, NF-*κ*B signaling pathway is the most common involved pathway in the inflammatory response [37, 38]. NF-*κ*Bp65, one of the NF-*κ*B subunits, is inactivated at normal physiological conditions and is bound to the inhibitor of kappa B (I*κ*B) proteins in the cytosol. However, when it is stimulated by inflammatory stimuli, such as viruses and bacterial toxins LPS, IKK first phosphorylates and degrades I*κ*B, NF-*κ*B begins to be activated, and NF-*κ*B is dissociated from the complex and transferred to the nucleus. In this study, we found that GLGZG markedly inhibited LPS-induced NF-*κ*B activation in BV2 cells by decreasing the phosphorylation level of I*κ*B*α*, NF-*κ*Bp65 nuclear level, and IKK induced by LPS stimulation. Furthermore, it is proved that the classical NF-*κ*B pathway mediates not only microglial activation but also neuron death [39]. Our previous experiment found that GLGZG produces its protective effect by inhibiting the apoptosis of neurons. And in this report, our data support that GLGZG suppressed microglial activation via inhibiting NF-*κ*B signaling pathways.

PI3K/Akt is an important regulator of inflammation and is reported to play an important role in negatively regulating LPS-induced IL-6 and TNF-*α* production from bone marrow macrophages [40]. And it is involved in NF-*κ*B-mediated neuroinflammation. Activation of Akt takes part in regulating proinflammatory responses in microglia by modulating NF-*κ*B signaling pathways [41]. Our previous study showed that the neuroprotective effects of GLGZG are achieved by activating the PI3K/Akt pathway [42]. In the present study, we found that GLGZG activates the phosphorylation of Akt induced by LPS in BV2 microglia. These results are in accordance with the fact that PI3K/Akt is a potent suppressor of inflammatory response in monocytes/macrophages [41, 43, 44]. Therefore, these results suggested that GLGZG might specifically suppress neuroinflammatory responses and inhibit NF-*κ*B signaling pathways in activated BV2 cells via the activation of the Akt signaling pathways.

Activated microglia release NO and proinflammatory cytokines and induce neuroinflammation, which are known to damage neurons [45]. Next, we aimed to examine whether GLGZG treatment protected against activated microglia-mediated neuroinflammation. As shown in results (Figure 9(a)), GLGZG significantly improved cell viability in LPS treatment-induced HT-22 cells. Thus, these results suggest that GLGZG inhibition of microglial activation could prevent injury to neurons.

## 6. Conclusions

To conclude, findings from this study illuminated that GLGZG could attenuate the inflammatory response of LPS-activated microglia via NF-*κ*B and Akt signaling pathways and protect neurons against activated microglia-mediated neuroinflammation. The study suggests that GLGZG may be a potential therapeutic agent for neuroinflammation.

## Figures and Tables

**Table 1:** Primers used for quantitative real-time PCR analysis.

Gene	Forward primer	Reverse primer
CD32	5′-AATCCTGCCGTTCCTACTGATC-3′	5′-GTGTCACCGTGTCTTCCTTGAG-3′
CD86	5′-GACCGTTGTGTGTGTTCTGG-3′	5′- GATGAGCAGCATCACAAGGA -3′
CD206	5′-CAAGGAAGGTTGGCATTTGT-3′	5′-CCTTTCAGTCCTTTGCAAGC-3′
Arginase-1	5′-TCACCTGAGCTTTGATGTCG-3′	5′-CTGAAAGGAGCCCTGTCTTG-3′
Ym1	5′-CAGGGTAATGAGTGGGTTGG-3′	5′-CACGGCACCTCCTAAATTGT-3′
IL-1*β*	5′-ATGACCTGTTCTTTGAGGCTGAC-3′	5′-CGAGATGCTGCTGTGAGATTTG-3′
IL-6	5′-GACCAAGACCATCCAACTCATC-3′	5′-ACATTCATATTGCCAGTTCTTCGTA-3′
IL-10	5′-CCAAGCCTTATCGGAAATGA-3′	5′- TTTTCACAGGGGAGAAATCG-3′
TNF-*α*	5′-ATGAGCACGGAAAGCATG-3′	5′-TACGGGCTTGTCACTCGAGTT-3′
iNOS	5′-CAAGCACCTTGGAAGAGGAG-3′	5′-AAGGCCAAACACAGCATACC-3′
GAPDH	5′- AGCCCAGAACATCATCCCTG-3′	5′-AGCCCAGAACATCATCCCTG-3′

## Data Availability

The datasets used and/or analyzed during the current study are available from the corresponding author upon reasonable request.
